# Criteria for enhancing mucus transport: a systematic scoping review

**DOI:** 10.1186/s40248-018-0127-6

**Published:** 2018-07-06

**Authors:** Alison Pieterse, Susan D. Hanekom

**Affiliations:** 1 Department of Physiotherapy, Tygerberg Hospital, Cape Town, South Africa; 2 Division of Physiotherapy Department of Health and Rehabilitation Sciences Faculty of Medicine and Health Sciences, University of Stellenbosch, 4th Floor Education Building Tygerberg, PO Box 241, Cape Town, 8000 South Africa

**Keywords:** Mucus transport, Airway mucus clearance, Scoping review

## Abstract

**Background:**

Uncertainty exists regarding the physiological basis of physiotherapy strategies to facilitate mucus clearance. The aim of this review was to describe the physiological factors and intrinsic conditions that facilitate airway mucus transport.

**Method:**

A scoping review was performed. A systematic literature search of six databases was executed. Eligibility criteria were applied by two researchers to reach the aim of the review. Papers were identified independently by two reviewers on title, abstract and full-text level. Any discrepancies were discussed with a third reviewer.

**Results:**

The search identified 35 papers published between 1975 and 2015. These differed significantly in terms of outcome measures, measurement techniques and methodologies and included animal studies, laboratory investigations, and the use of small human samples. Nine key factors influencing mucus transport were identified. These include: temperature and humidity, bronchial perfusion, ATP, forced expiratory technique and cough, generation of oscillation, ventilation patterns/airflow, epithelial differences, mucus properties and positioning.

**Conclusion:**

This review provides a framework for factors/conditions influencing mucus transport. Existing physiotherapy strategies for augmentation of airway mucus clearance can now be evaluated against the framework and new modalities informed.

**Electronic supplementary material:**

The online version of this article (10.1186/s40248-018-0127-6) contains supplementary material, which is available to authorized users.

## Background

Diagnosis of tuberculosis is a critical component of the WHO End TB Strategy [1]. However, obtaining a sputum sample in unproductive PTB (pulmonary tuberculosis) subjects remains challenging [2]. Improved non-invasive sputum collection interventions have the potential to increase diagnostic performance using available laboratory testing when compared with costly new diagnostic methods [2].

Efficient mucus clearance is needed for respiratory health and requires a coordinated system of epithelial water and ion transport, mucin synthesis and secretion, cilia action and cough [3, 4]. Therefore the regulation of these processes influences mucus clearance or transport.

Diseases which result in excessive or chronic secretion retention have prompted the investigation of numerous physiotherapy techniques and devices for the purpose of mucus clearance [5–11]. Physiotherapy techniques used for secretion clearance include positioning, percussion, vibration and shaking, breathing techniques, autogenic drainage and active cycle of breathing technique [6, 7]. Respiratory devices, namely positive expiratory pressure, high frequency chest wall oscillation, oral high frequency oscillation, intrapulmonary percussive ventilation, incentive spirometry, flutter, acapella, cornet, and the relatively new addition lung flute, are some examples of devices which have been used to augment clearance [7, 8, 10, 11].

Limited data is available on the effectiveness of various physiotherapy strategies and there is uncertainty regarding their physiological basis. The initial phase of research development includes preclinical trials which provide evidence of key individual components and the aim is to collect data supporting safety and indicating the potential usefulness of a new drug, procedure or medical intervention. This phase is then followed by phase 0 trials which are exploratory in nature and conducted in small human samples to confirm that the intervention, in fact, produces the expected or desired results in humans before proceeding to further larger scale clinical trials.

To inform the development of a framework of factors/conditions influencing mucus transport we completed a systematic scoping review of the literature. The aim was to systematically identify literature and describe physiological factors and intrinsic conditions which facilitate airway mucus transport. The secondary aims were to 1) describe the scope of the existing research; 2) map the research development phase in which the research was conducted; and 3) describe the outcome measures used. The aforementioned information was used to identify potential research gaps and clarify the potential for conducting a meta-analysis using the available data.

## Review process

The systematic scoping review was performed following the framework described by Arksey and O’Malley [12]. Before commencing this review, the Cochrane Library database was searched to ensure that no similar reviews have been published. The following databases were searched from database inception to March 2018: Medline, Scopus, Web of Science, CINAHL, Science Direct and Google Scholar (Refer to additional material for search strategies in Additional file 1: Addendum A). Papers were assessed on title, abstract and full-text level by two independent reviewers. In addition reference lists of included studies were screened independently by two reviewers. It was not necessary to contact authors requesting clarity of studies.

Papers were included if written in English, experimental (*in vitro* and *in vivo*) study designs; participants- humans over the age of 18 and animals- mammals; reported on physiological conditions investigated to optimise mucus transport or interventions/equipment to optimise mucus transport. Only papers which included the following study outcome were included: sputum volume expectorated, sputum displacement, sputum velocity, tracheal mucus clearance rate.

Papers were excluded if reporting on: mucus transport and pharmaceutical/pharmacology/pharmacodynamics; suction or subglottic secretion drainage or nasal mucociliary clearance; methods for studying mucus/mucociliary clearance models for evaluation; mucus transport in pulmonary diseased states or in systems other than the airway/respiratory system.

Data were extracted independently by two reviewers into a purposefully designed excel spreadsheet under the following headings: author, year of publication, specimen type, number of participants or model, physical factor/intervention, method of measurement, outcome measures and main findings.

## Results

**Fig. 1:**
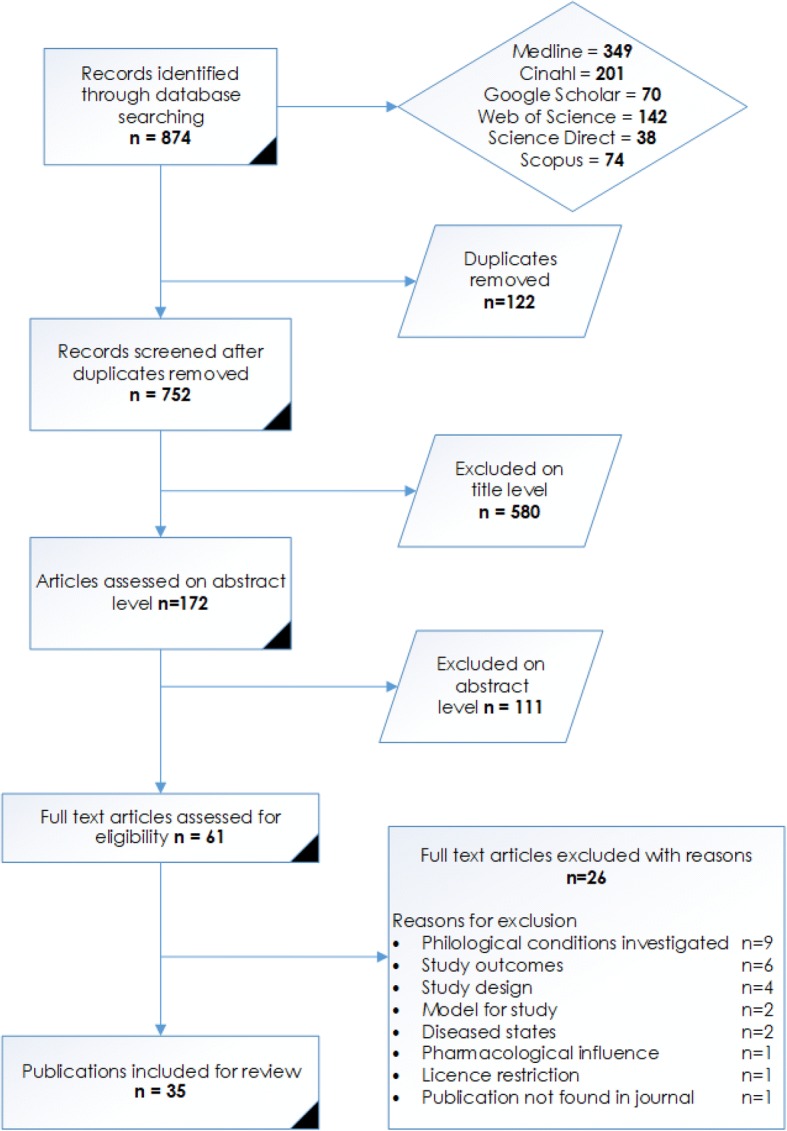
Review process flow diagram

The flow of information through the different phases of this review is represented in the review process flow diagram, Fig. 1 (From the PRISMA Group - [13]). The search yielded 874 papers and after the review process 35 were included in this scoping review (Refer to Fig. 1).

A brief description of the 35 papers which met the inclusion criteria can be found in Table 1. Papers were organized into pre-clinical trials and phase 0 trials.

**Table 1:** Papers included in the review

Author	Year	Phase			Physiological factor or intervention
		Preclinical	Phase 0	PC + 0	
Agarwal M, King M and Shukla JB	1994	×			Epithelial differences
Bennett WD; Foster WM; Chapman WF	1990		×		Forced expiratory technique and cough
Button B; Picher M; Boucher RC	2007	×			ATP
Diesel DA; Lebel JL; Tucker A	1991	×			Temperature and humidity
Eckerbom B; Lindholm CE; Mannting F	1991	×			Temperature and humidity
Freitag L; Kim CS; Long WM; Venegas J; Wanner A	1989	×			Generation of oscillation
Freitag, L., Long, W.M., Kim, C.S.	1989	×			Generation of oscillation
Gatto LA; Houck BM	1989	×			Epithelial differences
Gross, D., Zidulka, A., O’Brien, C., Wight, R., Fraser, R., Rosenthal, L., King, M.	1985	×			Generation of oscillation
Kilgour E; Rankin N; Ryan S; Pack R	2004	×			Temperature and humidity
Kim, C.S., Greene, M.A., Sankaran, S.	1986	×			Ventilation patterns/Air flow
Kim, C.S., Iglesias, A.J.	1987	×			Ventilation patterns/Air flow
King M	1987	×			Mucus properties
King M, Phillips DM, Gross D, Vartian V, Change HK and Zidulka A	1983	×			Generation of oscillation
King, M., Phillips, D.M., Zidulka, A., Chang, H.K.	1984	×			Generation of oscillation
King M, Zahm JM, Pierrot D, Vaquez-Girod S and Puchelle E	1989	×			Mucus properties
King M; Zidulka A; Phillips DM; Wight D; Gross D; Chang HK	1990	×			Generation of oscillation
Li Bassi G; Saucedo L; Marti JD; Rigol M; Esperatti M; Luque N; Ferrer M; Gabarrus A; Fernandez L; Kolobow T	2012	×			Ventilation patterns/Air flow
Li Bassi G; Zanella A; Cressoni M; Stylianou M; Kolobow T	2007	×			Positioning
Lippmann M, Albert RE, Yeates DB, Berger JM, Foster WM, Bohning DE	1975	×			Temperature and humidity
Martí, Joan Daniel; Li Bassi, Gianluigi; Rigol, Montserrat; Saucedo, Lina; Ranzani, Otavio Tavares; Esperatti, Mariano; Luque, Nestor; Ferrer, Miquel; Vilaro, Jordi; Kolobow, Theodor; et al.	2013	×			Generation of oscillation
Mortensen J; Jensen C; Groth S; Lange P	1991		×		Forced expiratory technique and cough
Piccin VS; Calciolari C; Yoshizaki K; Gomes S; Albertini-Yagi C; Dolhnikoff M; Macchione M; Caldini EG; Saldiva PH; Negri EM	2011	×			Ventilation patterns/Air flow
Puchelle E; Zahm JM; Jacquot J; Pierrot D	1989	×			Temperature and humidity
Radford, R., Barutt, J., Billingsley, J.G.	1982			×	Generation of oscillation
Ragavan AJ; Evrensel CA; Krumpe P	2010	×			Generation of oscillation
Rubin EM; Scantlen GE; Chapman GA; Eldridge M; Menendez R; Wanner A	1989	×			Generation of oscillation
Sears PR, Yin W-N, Ostrowski LE	2015	×			Temperature and humidity
Tatkov, S., Pack, R.J.	2011	×			Generation of oscillation
Trawöger R; Kolobow T; Patroniti N; Forcier K	2002	×			Ventilation patterns/Air flow
Volpe MS; Adams AB; Amato MBP; Marini JJ	2008	×			Ventilation patterns/Air flow
Wagner, E.M, Foster, W.M.	1996	×			Bronchial perfusion
Wong, J.W., Keens, T.G., Wannamaker, E.M., (...), Levison, H., Aspin, N.	1977		×		Positioning
Yang TQ; Majima Y; Guo Y; Harada T; Shimizu T; Takeuchi K	2002	×			Epithelial differences
Zahm JM; Pierrot D; Vaquez-Girod S; Duvivier C; King M; Puchelle E	1989	×			Mucus properties

**Preclinical study:**

Research using animals to find out if a drug, procedure, or treatment is likely to be useful. Preclinical studies take place before any testing in humans is done (https://www.cancer.gov/publications/dictionaries/cancer-terms?cdrid=44517 accessed 18/09/17)

A study to test a drug, a procedure, or another medical treatment in animals. The aim of a preclinical study is to collect data in support of the safety of the new treatment. Preclinical studies are required before clinical trials in humans can be started (http://www.medicinenet.com/script/main/art.asp?articlekey=5019 accessed 18/09/17)

Laboratory test of a new drug or a new invasive medical device on animal subjects; conducted to gather evidence justifying a clinical trial (http://www.thefreedictionary.com/preclinical+trial accessed 18/09/17)

**Phase 0 clinical trial:**

Even though phase 0 studies are done in humans, this type of study isn’t like the other phases of clinical trials. The purpose of this phase is to help speed up and streamline the drug approval process. Phase 0 studies are exploratory studies that often use only a few small doses of a new drug in a few patients. Phase 0 studies help researchers find out whether the drugs do what they’re expected to do. This process may help avoid the delay and expense of finding out years later in phase II or even phase III clinical trials that the drug doesn’t act as expected to based on lab studies (https://www.cancer.org/treatment/treatments-and-side-effects/clinical-trials/what-you-need-to-know/phases-of-clinical-trials.html - accessed 20/09/17)

Preclinical trials *n* = 31 (88%) including laboratory designs with simulated environments, *in vitro* - excised tracheas and cell cultures and *in vivo* animal laboratory interventions looking at fundamental mechanisms.

Phase 0 trials *n* = 3 (9%) included small human samples, providing preliminary information for further development of clinical modalities.

One study combined both preclinical and phase 0 trials *n* = 1 (3%) which demonstrates the next level of research translation.

### Publication timeline distribution

The 35 papers included in the review span over several decades from 1975 to 2015, with a spike in activity between 1989 and 1991.

### Outcome measures

A multitude of outcome measures were utilised. These included representations of velocity, distance, mass and percentage clearance.

**Table:** 

Increase Mucus Transport	Decreased Mucus Transport	No effect on Mucus Transport
Physiological factors • Increase temperature 0 - 37 °C [4] • “tidal breathing”/Cyclic compressive stress to lung [14] • Increase in peak inspiratory flow (PIF) [15] • Increase in expiratory – inspiratory flow difference (E-I) (achieved by increased duty cycle (Ti/Ttot) and PEEP) [15, 16] • Expiratory flow bias [15] and increased PEFR [17] • Cough - Cough clearance (CC) increases with: decreased viscosity, spinnability and adhesivity of mucus [18]; more elastic cohesive mucus [19]; increased peak flow rate [20] and increased cross-sectional area occupied by longitudinal loss of cilia [21]; HFO 25 – 68 Hz [19]; Head-up position 0 - 45° [19] Interventions/equipment • Heat moisture exchanger (HME) presence [22] • High frequency chest wall compression (HFCW) or oscillation (11-15 Hz, peaking at 13 Hz) [23–27] • Hard manual rib cage compression (resulting in increase peak expiratory flow (PEF) and increase mean expiratory flow (MEF) and increase in MEF – MIF (mean expiratory flow – mean inspiratory flow) with head up 20-30° [28] • Percussion energy on chest wall of 25-30 Hz – optimal with head down 60° and 60° head up [29] • High-frequency oscillation with expiratory peak flow bias [25, 30] • HFO with head up 0-45° [19] • Head down tilt of 5° [31] • Controlled coughing – short-term benefit [32]	Physiological factors • Decreased temperature 37° - 25 °C [4, 33, 34] • Lower air humidity (9 g water/m^3^) [35] • Bronchial blood flow stopped [36] • Increased mucus viscosity [17] • Diminished cough: CC decreases with increased viscosity [18] and increased elasticity more than viscosity [20]	Physiological factors • Head flexion or extension [37] Interventions/equipment • Commercial oscillator (40 Hz) [27] • Ventilation at low volume, high volume, high pressure [38] • Positioning upright or head down tilt 25° [39] • Forced expiration [40]

### Factors investigated

Nine categories emerged: temperature, humidity and heat; bronchial perfusion; ATP; forced expiratory technique (FET) and cough; generation of oscillation; ventilation patterns/airflow; epithelial differences; mucus properties and positioning.

#### Temperature, humidity and heat

Six papers [4, 22, 33–35, 41] reported on the effect of temperature or humidity on the movement of mucus (Table 2). While different measurements were used in the various papers, mucus transport decreased with decreasing temperature and increased with increasing temperature and humidity.

**Table 2:** Temperature and humidity

Publication	Human	Animal/Lab	Temperature range	Humidity	Tracheal mucus transport measurement method	Effect on mucus Transport
Diesel et al. 1991		x	35-39.5 °C		Timing of particles as they travelled the 1-cm distance from 1 grid bar to the next	↓ with ↓ temp
Eckerbom et al. 1991		x		HME vs no HME	Bronchoscopy and gamma camera	↑ with HME
Kilgour et al. 2004		x	30-37 °C	100% relative humidity	Timing movement of reflective particles in the mucus across a calibrated eyepiece graticule	↓ with ↓ temp
Lippman et al. 1975		x	−10° - 30 °C		Rectilinear scanner and the calculated time required for a bolus of tagged particles to move a fixed distance along the trachea	↓ with ↓ temp
Puchelle et al. 1989		x		Absolute humidity of 9 g water/m^3^ vs 30 g water/m^3^	Ciliary transport capacity analysed using the frog palate technique and transport velocity assessed by measuring transit time to pass over a 2 mm portion of the palate	↓ with ↓ humidity
Sears et al. 2015		x	0 - 37 °C 37 - 25 °C 24.5 – 39.5 °C		Microscopy and reviewing videos and tracking the motion of individual particles.	↑ with ↑ temp ↓ with ↓ temp

In a laboratory tracheal model, Kilgour et al. [33] found that a decrease in air temperature from 37° to 34/30° at 100% relative humidity resulted in a decrease in ciliary beat frequency (CBF) and a reduction in mucus transport velocity. This potentially produced mucociliary failure and epithelial damage.

Diesel et al. [41] also established a similar direct relationship between mucosal temperature and tracheal mucus velocity within mucosal temperature of 35-39.5° in the excised tracheas of cold-exposed calves.

Eckerbom et al. [22] evaluated the presence of a heat moisture exchanger (HME) for the purpose of moistening inhaled air and noted a non-significant increase in mucociliary transport velocity in the HME group and tracheal desiccation in the control group.

Lippmann et al. [34] investigated the ambient temperature and humidity in control tests on three donkeys over a 13 month period and temperatures varied from − 10 to 30 °C. The tracheal transport rate decreased by approximately 1.8%/°C decrease in temperature.

In anaesthetised dogs, Puchelle et al. [35] examined the influence of inspired absolute humidity of 9 g water/m^3^ versus 30 g water/m^3^ on mucus transport capacity and demonstrated that a lower air humidity decreased mucus transport rate. This finding was positively and significantly correlated to mucus spinnability and the differences in mucus spinnability attributed to changes in air humidity.

Sears et al. [4] examined the effect of temperature on CBF and mucociliary transport by increasing smoothly from 0 to 37 °C; decreasing smoothly 37 - 25 °C and changing in steps 24.5 – 39.5 °C. CBF increased with increasing temperature and mucociliary transport increased in parallel with the increase in CBF. CBF and mucociliary transport decreased with decreasing temperature.

#### Bronchial perfusion

Only one publication was included in this section. In a sample of 8 anaesthetised sheep, Wagner and Foster [36] found significantly diminished airway clearance of particles when bronchial blood flow was stopped when compared with a control (unaltered bronchial blood flow).

#### ATP

This section only included one paper investigating ATP release and mucociliary transport by human airway epithelia [14]. The data suggests that cyclic compressive stress, mimicking normal tidal breathing may regulate ASL volume in the normal lung.

#### FET and cough

Mortensen et al. [40] investigated slow inspiration and forced expirations or forced inspiration and slow expiration and found that repeated dynamic compressions associated with forced expiration did not affect bronchial clearance in healthy subjects or a small sample of patients with chronic bronchitis. Bennett et al. [32] found that in healthy non-smoking subjects acting as their own controls, a controlled coughing intervention increased the rate at which the radiolabeled particles were cleared from the bronchial airways at one and two hours but no difference in retention of particles after twenty-four hours when compared with the same participant on the control day.

#### Generation of oscillation

This section examines the application of an external force on the chest wall [23–29] and high-frequency oscillating airflow [19, 24, 25, 30, 42, 43] (Table 3).

**Table 3:** Generation of oscillation

Publication	Animal/Lab	Human	Frequency	Chest wall			Airflow		Tracheal orientation
				HFO/CW	Percussion	MRCC	HFO	HFO/AO	
King et al. 1983	x		3-17 Hz	x					Horizontal
King et al. 1984	x		13 Hz CW	x				x	Horizontal
			13 Hz, 17-20 Hz AO						
Gross et al. 1985	x		13 Hz	x					Horizontal
Ruben et al. 1989	x		13 Hz and 40 Hz	x					Horizontal
Marti et al. 2013	x		Hard-brief and soft-gradual			x			20.9° ± 4.5°
Radford et al. 1982	x	x	0-100 Hz		x				−60°- + 60°
Tatkov et al. 2011	x		20 Hz or 14 / 20 Hz				x		−15° or Flat
King et al. 1990	x		13 Hz	x				x	Horizontal
Freitag et al. 1989	x		14 Hz					x	Horizontal −15°
Freitag et al. 1989	x		15 Hz				x		Horizontal
Ragavan et al. 2010	x		25-68 Hz				x		0°,+ 15°,+ 30° and + 45°

*HFO/CW* High frequency oscillation applied to chest wall

*MRCC* Manual rib cage compression

*HFO* High frequency oscillating airflow

*HFO/AO* High frequency oscillating airflow applied to the airway opening

King et al. [23] demonstrated that high-frequency chest wall compression/oscillation (HFO/CW) increased tracheal mucus clearance rate (TMCR), with the enhancement of clearance most pronounced in the range of 11-15 Hz, peaking at 13 Hz. King et al. [24] then further found that high-frequency oscillation at the airway opening (HFO/AO) did not improve tracheal mucus clearance (76% of control) compared with spontaneous breathing, whereas HFO/CW at 13 Hz enhanced tracheal mucus clearance (240% of control).

The following year, Gross et al. [26] also used a similar spontaneously breathing population and measurement technique and found that HFO/CW at a frequency13 Hz significantly enhanced peripheral mucociliary clearance.

Ruben et al. [27] used two chest wall oscillators to investigate the effect on central airway mucociliary clearance. The commercial oscillator was used at its minimum frequency of 40 Hz and had no effect on tracheal mucus velocity (TMV) while the experimental oscillator which produced a frequency of 13 Hz significantly increased TMV independent of the baseline TMV.

Marti et al. [28] investigated the effects of two variations of manual rib cage compression on expiratory flow and mucus clearance during prolonged mechanical ventilation in pigs. The researchers found that hard manual rib cage compression moved mucus towards the glottis with animals positioned 20-30° above horizontal. During Hard manual rib cage compression (MRCC), the peak expiratory flow (PEF) and mean expiratory flow (MEF) increased significantly and the MEF-MIF difference was significantly increased by the hard manual rib cage compression as opposed to no treatment or soft manual rib cage compression. Mucus moved towards the lungs with no treatment and soft manual rib cage compression.

Radford et al. [29] demonstrated that percussion energy applied to the chest wall of dogs and humans altered flow rates and pattern and percussion energy at 25-35 Hz appeared to be the most favourable frequency range for mucociliary transport. The researcher noted the greatest increase in transport rate at tracheal orientation of 60° head down.

Tatkov et al. [42] used 2 different tracheal preparations to investigate the effect of high-frequency oscillation (HFO) on mucus flow. Within this study, 2 different methods were used to measure mucus-transport velocity. Symmetrical waveform HFO at 20 Hz and amplitude of 50cmH_2_O, applied to an intact tracheal preparation in the presence of a thick layer of artificial mucus with the trachea cephalad-end-down tilt 15° resulted in an increased mucus transport velocity whereas HFO at 14/20 Hz in an open, flat mounted tracheal experiment, did not significantly alter that velocity.

King et al. [25] found that tracheal mucus clearance (TMCR) was significantly increased with HFO/CW of 13 Hz compared with HFO/AO, however, TMCR with HFO/AO was greater with an expiratory peak flow bias (expiratory peak flow > inspiratory peak flow) than symmetrical flow or inspiratory bias (inspiratory peak flow > expiratory peak flow).

Freitag et al. [30] examined the effect of posture (prone and right side lying) and HFO airflow bias on mucus movement where the artificial mucus used was comparable to that of natural mucus. Mucus clearance with HFO of 14 Hz with expiratory bias at the airway opening of ventilated sheep was not significantly enhanced by head-down tilt of 15°. However, clearance in head-down tilt alone was significantly improved with the addition of HFO with expiratory bias. No clearance occurred with inspiratory biased flow in head-down tilt position. In another study performed by Freitag et al. [43] in sheep tracheas, the researchers reported that asymmetrical high-frequency ventilation at 15 Hz with expiratory biased flow profiles was able to move mucus towards the pharynx.

Ragavan et al. [19] found significant interactive influence among cough velocities, tracheal angles, simulant types and oscillations on mucus displacement during cough. The more elastic cohesive mucus simulant travelled significantly larger distances at all angles of tracheal inclination (horizontal, 15°, 30° and 45°), at all cough velocities, with or without airflow oscillations compared with the thinner mucus simulant. Superimposed flow oscillations (25-68 Hz) significantly increased the magnitude of mucus displacement for both types of simulant and the magnitude was significantly greater with higher tracheal inclination (head up) compared with the horizontal for both mucus preparations – both with and without oscillations [19].

#### Ventilation patterns/airflow

In this section examining airflow patterns, Volpe et al. [15] used a test-lung system to investigate the role played by ventilator patterns on secretion clearance and retention. Only peak inspiratory flow significantly correlated with centre-of-mass displacement and univariate analysis revealed that both expiratory – inspiratory flow difference (E-I difference) more so than expiratory/inspiratory flow ratio (E/I) were important correlates of mucus movement.

Li Bassi et al. [16] showed that in pigs in the semi-recumbent position the prolongation of the duty cycle (Ti/Ttotal) decreased the inspiratory flow rate which consequently increased the expiratory – inspiratory flow bias and promoted the movement of mucus towards the glottis. The suction technique was a potential confounder as prolonged mechanical ventilation and longer time since last tracheal aspiration were associated with a greater risk of mucus flow towards the lungs.

The use of intratracheal pulmonary ventilation (ITPV) demonstrated a slow then rapid cephalad movement of mucus distal to the tip of the endotracheal tube in a model trachea [44].

Piccin et al. [38] investigated the effects of various mechanical ventilation strategies on the mucociliary system and found that high-pressure ventilation decreased respiratory compliance and injury was demonstrated in the ciliated cells of the high-pressure group with a significantly decreased ciliary beat frequency after mechanical ventilation. Tracheal mucus clearance did not change significantly in the ventilated groups. All ventilated animals showed a reduction of mucus on tracheal epithelium compared to the control. They concluded that mechanical ventilation leads to lung and tracheal alterations leading to mucociliary dysfunction.

Kim et al. [17] found in a horizontal tube model that liquid layer transport speed (LLTS) increased with increasing peak expiratory flow rate and at the same peak expiratory flow rate, LLTS was higher with viscoelastic than viscous liquid. In the vertical tube model, at high values of peak expiratory flow rate, LLTR was comparable to that in the horizontal tube model. The results indicated that LLTS is mainly governed by the absolute value of the higher airflow and not by the E – I difference. Kim et al. [45] found transport speed in vertical tube model increased with increasing airflow rates for all test solutions but decreased rapidly with increasing viscosity of mucus.

#### Mucus properties

This section included mucus properties influencing mucus movement. Using a simulated cough machine, King et al. [46] found viscosity as the major independent variable relating to cough clearance. The researchers also found highly significant dependencies for spinnability and adhesivity. Cough clearance correlates inversely with both viscosity and spinnability and shows a residual negative relationship with adhesivity. There was no significant correlation with either elastic modulus or relaxation time. Using similar system as the above study, Zahm et al. [18] found that the addition of a sol phase simulant significantly decreased the adhesivity and wettability of the gel mucus simulants and this was associated with a marked enhancement of cough clearance irrespective of the viscoelastic properties of the gel mucus simulants. In the absence of sol phase simulant, a significant negative relationship was found between viscosity and cough clearability of mucus simulants.

King [20] found that for any initial depth of mucus or rheological state, cough clearance index increased with increasing peak flow rate. Cough clearance (in a simulated cough machine) was impeded more by elasticity than viscosity and an inverse relationship was found between viscoelasticity and particle transport.

#### Epithelial differences

Three papers were included in this section. A study by Yang et al. [47] indicated that the degree of loss of cilia contributes to mucociliary deceleration. The mucus-depleted frog palate epithelium shares characteristics with the mammalian airway and in this study was subjected to mechanical damage. Under these conditions, the mucociliary transport rate (MTR) of a small amount of mucus was significantly decreased compared with that of a large amount of mucus. There was no difference in MTR between a small/large amount of mucus in the undamaged frog mucosa. Mucosal damage was regular which is unrealistic clinically and the longevity of the preparations was not determined.

Agarwal et al. [21] created longitudinal channel grooves, representing spacing between arrays of cilia, in a simulated cough machine. Mucus gel transport increases as the cross-sectional area occupied by the channel grooves increases.

Gatto et al. [37] found that during extension of the head of a rat, the length of the trachea increased by 50% without a change in diameter. Extension caused surface epithelial cells to elongate longitudinally and to decrease in height. These changes were greater in ciliated cells than in mucus-containing cells. However, the mucociliary clearance rate did not change with head flexion or extension.

#### Positioning

This section includes two studies with the primary and sole intervention being positioning. Wong et al. [39] found in a small sample of healthy spontaneously breathing subjects that there was no significant difference in the rate of mucus movement between two positions. Sixteen intubated and ventilated sheep were studied by Li Bassi et al. [31] in two positions of which head down promoted mucus movement to the glottis.

## Discussion

The review identified nine factors which have been investigated as influencing mucus movement. These include: temperature and humidity, bronchial perfusion, ATP, FET and cough, generation of oscillation, ventilation patterns/airflow, mucus properties, epithelial differences and positioning.

Mucus transport has been a field of research interest for more than 30 years. The review provides an overview of the factors which affect mucus transport. While there is great variability of outcome measures documented in the body of literature, all measurements reported are indicators of movement. The majority of the data is categorized as preclinical trials, the findings of which do not necessarily translate into clinical practice, however, provide evidence of fundamental elements influencing mucus transport.

We hypothesize, based on the data presented, that, in order to facilitate mucus transport, a single device will not suffice. A more holistic approach such as the development of a protocol which can include the nine factors identified in this review should be considered. We propose that factors be categorised into 1) environmental conditions and 2) patient-related conditions.

Environmental conditions identified in the review include temperature and humidity, bronchial perfusion, ATP, and epithelial differences. Exposure to temperatures below body temperature, low air humidity and diminished bronchial blood flow adversely affect mucus transport. Colder temperatures may adversely affect mucus properties and epithelial integrity. The mechanism responsible for decreased airway clearance when bronchial perfusion was stopped is unclear; however bronchial circulation may influence nutrient flow, airway wall temperature and humidity and release of mediators associated with tissue ischaemia [36]. Maintenance of airway surface liquid (ASL) volume is regulated by luminal ATP, keeping it at optimal levels of mucociliary clearance. Button et al. [14] found that in normal airway epithelia, cyclic compressive stress-induced increase in ASL ATP concentration was sufficient to induce purinoceptor-mediated increases in ASL height and mucociliary clearance.

Patient-related conditions identified in the review include FET and cough, generation of oscillation, ventilation patterns /airflow, mucus properties and positioning. A recurring finding in the literature is HFO of 13 Hz being referred to as the optimal frequency for mucus transport. Interesting to note was that HFO of chest wall was found to promote mucus clearance to a greater extent than at the airway opening. However, HFO/AO with expiratory bias enhanced clearance while inspiratory bias does not favour clearance. This data suggests that applying HFO to the chest wall or using a device to facilitate HFO at the air opening during forced expiration has the potential to facilitate mucus clearance. While higher frequencies of 25 – 68 Hz [19, 29] and hard MRCC [28] have also reported to increase mucus transport, unfortunately, these vibration and percussion frequencies are usually outside manual capability in the clinical setting.

Contrary to earlier studies investigating postural drainage positions as a technique to facilitate mucus clearance [48], the data suggests that positioning may not influence mucus transport independently [31, 39], however combined with manual physiotherapy techniques, enhances mucus transport. Tracheal orientation/gravitational effects were a confounding factor or intentionally incorporated into multiple interventions described in the sections above.

Ragavan et al. [19] found HFO in a higher tracheal inclination favoured mucus displacement and Marti et al. [28] also made use of head up position for MRCC, whereas Radford et al. [29] found the greatest increase in mucus transport with percussion in head down position. Recommendations in terms of therapeutic modalities include hard manual rib cage compressions, high-frequency oscillations and cough, all in head up 30-45°. The trachea down 5° position appeared beneficial for mucus drainage or clearance.

Peak expiratory flow or expiratory flow bias is considered a key contributor promoting mucus transport by numerous researchers in preclinical studies. Kim et al. [45] attributed increases in mucus transport to increasing airflow rates for all test solutions. Similarly, Marti and colleagues found mucus movement toward the glottis with hard MRCC corresponded with significant increases in peak expiratory flow and mean expiratory flow, with significant mean expiratory and inspiratory flow difference [28]. Both Kim et al. [17] and Volpe et al. [15] concur, emphasising the value of an increased peak expiratory flow rate (PEFR). Volpe et al. [15] identified an expiratory flow bias (PEFR>PIFR) with expiratory – inspiratory flow difference (E-I) being significant as correlated for mucus movement away from the lungs. The positive effect of expiratory flow bias on mucus clearance is also noted in combination with high-frequency airway oscillation [25, 30, 43].

It was interesting to find no evidence (Phase 0) to support controlled coughing and forced expiration as modalities to facilitate mucus transport. The mucociliary transport system includes cough clearance as a primary component. Based on the ventilation characteristics described above as promoting airway clearance, it is expected that the high flows and shearing forces generated with “huffing”/FET and coughing would affect airway clearance, however Mortensen's findings et al. [40] were contrary and Bennet's ones et al. [32] were unsustained.

King et al. [20, 46] and Zahm et al. [18] described a negative relationship between cough clearance and viscosity, viscoelasticity, spinnability and adhesivity. When investigating altering airflow patterns, Kim et al. [45] also found a decreased mucus movement with increasing viscosity of mucus. The results indicate that instructing a patient to cough will not independently facilitate the production of a sputum sample.

The results of the review must be interpreted with caution. Many of the animal trials had limitations such as those highlighted by Hooijmans et al. [49]. While it was challenging to interpret the data within a clinical context, the review highlights the complexity and multifactorial conditions needed for mucus transport. The data can now be used to develop a more comprehensive protocol to facilitate mucus transport.

## Conclusion

The nine categories of influence identified affect the respiratory system by means of external forces or within the airway demonstrating extensive potential in terms of approaches to contributing to mucus transport. The current available level of investigations would require further development before translation into clinical practice. Due to the heterogeneity of data in terms of participants or experimental models, methodologies, measurement techniques and outcome measures used, the act of rigorous research synthesis was not undertaken. Rather an overview of existing evidence was presented, regardless of methodological quality.

Underpinning the physiology of airway mucus transport and the characteristics of interventions which facilitate airway clearance is an essential step in the process of defining populations which would most benefit from these non-surgical, non-pharmaceutical treatment strategies. This review can be used as a framework to evaluate existing physiotherapy interventions and inform future modalities in order to optimally augment airway mucus clearance.

## Additional file

Additional file 1: Database Search Strategy. (DOCX 17 kb)

## Abbreviations

ATP: An extracellular nucleotide
CBF: Ciliary beat frequency
CC: Cough clearance
E – I: Expiratory – inspiratory flow difference (E-I)
FET: Forced expiratory technique
HFCW: High frequency chest wall
HFO: High frequency oscillating airflow
HFO/AO: High frequency oscillating airflow applied to the airway opening
HFO/CW: High frequency oscillation applied to chest wall
HME: Heat moisture exchanger
MEF: Mean expiratory flow
MEF – MIF: Mean expiratory flow – mean inspiratory flow
MRCC: Manual rib cage compression
PEEP: Positive end expiratory pressure
PEF: Peak expiratory flow
PEFR: Peak expiratory flow rate
PEFR > PIFR: Expiratory flow bias
PIF: Peak inspiratory flow
Ti/Ttot: Duty cycle = total inspiratory time as a fraction of respiratory cycle time
