# Supplementary material for: Criteria for enhancing mucus transport: a systematic scoping review
Source: Multidiscip Respir Med. 2018 Jul 6;13:22. doi: 10.1186/s40248-018-0127-6 (PMC6034335; doi:10.1186/s40248-018-0127-6)
Supplement: Supplementary file 1 — Database Search Strategy. (DOCX 17 kb) [file 40248_2018_127_MOESM1_ESM.docx]

**ADDENDUM A**

Each database has its own indexing and search functions (Search Strategies) and the databases used have therefore been grouped according to their search function characteristics.

- MeSH – CINAHL and Medline use key terms and Medical Subject Headings (MeSH) to classify papers and they allow terms to be combined.
- Key terms with limited combinations – Scopus, Web of Science and Science Direct databases also use key terms to classify papers however its functions have a limited ability to combine key terms.
- No limits on keywords allowed per search – Google Scholar

The strategy for searching each of the above-mentioned databases is as follows:

When using CINAHL and Medline, keywords (Keyword or concepts) describing the review question (interventions and outcomes) are to be mapped to the MeSH. Both keywords and MeSH will be used in the search, using appropriate combining terms (AND or OR). Appropriate truncation symbols were used.

When searching the Scopus and Web of Science database, keywords were used and combined using the OR function. Truncation symbols were used where possible.

When using Google Scholar a combination of keywords were searched.

**Search strategy for** **CINAHL**

Full site search

Search terms:

“mucus velocity”

“mucus displacement”

“tracheal mucus clearance rate”

“mobilization of mucus”

Search terms combined with “AND”

Search mode: Boolean/Phrase

MESH “mucociliary clearance” Combine with “AND” (search terms as described) above

Include All subheadings

Limits: Abstract available, academic journals

Refine search with “NOT” drug therapy, “NOT” pharmacology and “NOT” pharmacodynamics

**Search strategy for** **Medline**

Full site

Search terms:

“mucus velocity”

“mucus displacement”

“tracheal mucus clearance rate”

“mobilization of mucus”

Search terms combined with “AND”

Search modes: Boolean /Phrase

MESH “mucociliary clearance” AND (Search terms as described above)

“AND” Subheading Physiology

Limit: Abstract available and English language

Source type: Academic Journal

Subject: Major Heading – Mucociliary clearance

**Search strategy for Scopus**

Search terms in Title, abstract or keyword:

“mucus velocity”

“mucus displacement”

“tracheal mucus clearance rate”

“mobilization of mucus”

Search terms combined with “OR”

Exclude Subject Area: pharmacology, toxicology and pharmaceuticals and Limit to only English articles.

**Search strategy for Web of Science**

Search terms:

“mucus velocity”

“mucus displacement”

“tracheal mucus clearance rate”

“mobilization of mucus”

Search terms combined with “OR”

Refine search with:

Database search – “Web of Science Core Collection”

Research Domain - “Science and technology”

Research area limited to “Respiratory System” and Excluding “Pharmacology” and “Anatomy and Morphology”

Limit document type to – “article” and language to – “English”

**Search strategy for Science Direct**

Expert Search

Journals

Search terms:

“mucus velocity”

“mucus displacement”

“tracheal mucus clearance rate”

“mobilization of mucus”

Search terms combined with “OR”

Refine search with:

“All”

“All Sciences”

“Article”

“All Years”

Refine further selecting Publication Title: “Respiratory Medicine” and “Respiration Physiology”.

**Search strategy for Google Scholar**

Search: criteria for mucus transport mechanisms in the airways “mucus transport” AND “mucus clearance” –hyperinflation –pharmacology –nasal –children –mucolytic –bronchodilator

Includes all the words: criteria for mucus transport mechanisms in the airway

Exact phrase: “mucus transport” AND “mucus clearance”

Without the words: hyperinflation, pharmacology, nasal, children, mucolytic, bronchodilator

Exclude: patent, citations
